# Three new species of mealybug (Hemiptera, Coccomorpha, Pseudococcidae) on persimmon fruit trees (*Diospyros kaki*) in southern Brazil

**DOI:** 10.3897/zookeys.584.8065

**Published:** 2016-04-25

**Authors:** Vitor C. Pacheco da Silva, Mehmet Bora Kaydan, Jean-François Germain, Thibaut Malausa, Marcos Botton

**Affiliations:** 1 Plant Protection Graduate Program, Plant Protection Department, UFPel, Campus Universitário Capão do Leão s/n, Capão do Leão, Rio Grande do Sul, Brazil; 2 Imamoglu Vocational School, Çukurova University, 01330, Adana, Turkey; 3 Laboratoire de la Santé des Végétaux, Unité d’entomologie et plantes invasives, CBGP 755 avenue du campus Agropolis, CS30016, 34988 Montferrier-sur-Lez, Languedoc-Roussillon, France; 4 Institut Sophia Agrobiotech, UMR INRA, Université Nice Sophia Antipolis, CNRS, 400 Route des chappes, Sophia Antipolis, PACA, France; 5 Embrapa Grape and Wine, 515 Rua do Livramento, Bento Gonçalves, Rio Grande do Sul, Brazil

**Keywords:** Distribution, Neotropical Region, scale insects, taxonomy

## Abstract

Brazil has the greatest insect diversity in the world; however, little is known about its scale insect species (Hemiptera: Coccomorpha). Mealybugs (Pseudococcidae) have been found in at least 50% of persimmon orchards *Diospyros kaki* L. in the southern part of the country. In this study three new mealybug species on persimmon trees located in the Serra Gaúcha Region, RS, Brazil, namely, *Anisococcus granarae* Pacheco da Silva & Kaydan, **sp. n**., *Ferrisia kaki* Kaydan & Pacheco da Silva, **sp. n**. and *Pseudococcus rosangelae* Pacheco da Silva & Kaydan, **sp. n.** are described. In addition, an identification key for the genera occurring on fruit orchards and vineyards in Brazil is provided, together with illustrations and molecular data for the new species.

## Introduction

Southern Brazil is the third largest fruit-producing region in the country. It produces large amounts of temperate fruits, such as grape, apple, stone fruits and persimmon (Fachinello et al. 2011). Persimmon trees (*Diospyros kaki* L.) (Ebenaceae) were first cultivated in Brazil in the late 19^th^ century, but this crop expanded only after Japanese immigration, around 1920 (Neuwald et al. 2009). Persimmon is currently grown on about 9,000 ha and about 172,000 tons of fruits are produced annually, for domestic consumption and export (Fachinello et al. 2011). The São Paulo and Rio Grande do Sul states are the main producers of persimmon fruits in Brazil (Camargo-Filho et al. 2003). In Rio Grande do Sul, fruit production occurs mostly in the Serra Gaúcha Region, in which mealybugs (Hemiptera: Coccomorpha: Pseudococcidae) have been detected in at least 50% of production areas (Bavaresco et al. 2005), probably due to increases in insecticide application in recent years, leading to a decrease in the population of effective natural enemies.

Ten mealybug species have been recorded in association with persimmon trees worldwide: *Dysmicoccus brevipes* (Cockerell), *Hippeococcus wegneri* Reyne, *Maconellicoccus hirsutus* (Green), *Phenacoccus aceris* (Signoret), *Phenacoccus pergandei* Cockerell, *Planococcus citri* (Risso), *Planococcus kraunhiae* (Kuwana), *Pseudococcus cryptus* Hempel, *Pseudococcus longispinus* Targioni Tozzetti and *Pseudococcus viburni* (Signoret) (García et al. 2016).

Live mealybugs are small soft-bodied, sap-sucking insects with an oval, elongated to rounded body, often dorsoventrally compressed, pinkish to grayish in color, covered with a white powdery wax (the source of their common name) (Cox and Pearce 1983). They frequently have waxy filaments, those on the head being shorter than those close to the anus (Gimpel and Miller 1996). These filaments originate from the cerarii – groups of trilocular pores, generally with two conical setae and, in some groups, also auxiliary setae (Williams and Granara de Willink 1992) predominantly found along the margin. The family to which mealybugs belong is the second largest family in infraorder Coccomorpha, in terms of the number of species it contains, almost 2020 species, distributed in 260 genera (García et al. 2016). In the Neotropical Region, only 223 species, from 44 genera, have been recorded (García et al. 2016).

Pseudococcidae can be divided into two subfamilies: Pseudococcinae, characterized by the presence of: (i) apically knobbed tarsal digitules; (ii) claws without a denticle; (iii) antennae generally with eight or fewer segments; (iv) anal ring with setose-like spinules; and (v) absence of quinquelocular pores; and Phenacoccinae, characterized by: (i) setose tarsal digitules; (ii) claws with a denticle; (iii) antennae usually nine-segmented; (iv) anal ring with dome-shaped spinules on the outer ring; and (v) presence of quinquelocular pores (Hardy et al. 2008; Kaydan et al. 2015).

In total, 153 species of *Pseudococcus* (Westwood) have been identified worldwide, 30 of which have been recorded in the Neotropical Region. It can be subdivided into two informal groups according to the presence or absence of simple pores associated with each eye (Gimpel and Miller 1996) — present in *Pseudococcus maritimus* complex. It includes 33 species, 21 of which are present in the Neotropical Region (García et al. 2016), and is assumed to have originated from the New World, where some species, such as *Pseudococcus sociabilis* Hambleton, *Pseudococcus viburni* (Signoret) and *Pseudococcus maritimus* (Ehrhorn), are considered to be major pests of fruit crops and vineyards (Casco Mila 2012; Correa et al. 2012; Daane et al. 2012; Pacheco da Silva et al. 2014).

The genus *Ferrisia* Fullaway, which is of New World origin, includes 18 species, most (12 species) of which occur in the Neotropics (Kaydan and Gullan 2012). The species from this genus are easily separated from the other genera in the Pseudococcidae by the presence of robust dorsal enlarged tubular ducts opening to the exterior via an irregularly circular sclerotized area bearing one or more setae and, often, one or more minute pores (Gullan et al. 2010). Furthermore, the living insects have long glassy filaments produced by the enlarged tubular ducts, and, depending on the species, may have typical dorsal patterns formed by dark areas of cuticle not covered by white wax (Kaydan and Gullan 2012).

The genus *Anisococcus* Ferris is also believed to have originated from New World and to be closely related to *Ferrisia* on the basis of both molecular phylogenetic studies (Downie and Gullan 2004; Hardy et al. 2008; Gullan et al. 2010) and morphological studies, as it has minute discoidal pores associated with enlarged tubular ducts and oral collar tubular ducts (Kaydan and Gullan 2012). This genus is found exclusively in the Americas, where 11 species have been described, nine in the Nearctic region and two from the Neotropical Region (McKenzie 1967; Williams and Granara de Willink 1992).

Brazil has the greatest biodiversity of any country worldwide and 13% of all species (including animals, plants, fungi and other organisms) are found only in Brazil (Lewinsohn and Prado 2005). Insect diversity is also greater in Brazil than in any other country, with almost 100 thousand species recorded (almost 10% of all insect species worldwide) (Rafael et al. 2009). It has been estimated that almost 11% of hemipteran insects are present in Brazil. However, only 3.8% of mealybug species have been recorded in this country, although this should probably be regarded as an underestimate of the percentage actually present.

In Brazil, 530 species, from 20 families of the infraorder Coccomorpha have been recorded (García et al. 2016). In total, 78 species from 21 genera of Pseudococcidae have been detected in Brazil. The most numerous genera are *Dysmicoccus* Ferris (13) and *Pseudococcus* (13), followed by *Phenacoccus* Cockerell (10), *Nipaecoccus* Sulc (8), *Planococcus* (Ferris) (5) and *Ferrisia* (5) (García et al. 2016). Only one *Anisococcus* species, *Anisococcus parasitus* Williams and Granara de Willink, has been recorded from Brazil (Williams and Granara de Willink 1992).

Three new species of mealybugs sampled from persimmon orchards located in the Serra Gaúcha Region, Rio Grande do Sul, Brazil are described, and an identification key for the genera occurring in fruit orchards and vineyards in Brazil is provided, together with illustrations, molecular data and an identification key for the new species of *Anisococcus*, *Ferrisia* and *Pseudococcus* described here.

## Methods

Mealybugs were collected from persimmon orchards during the harvest period in the years 2013–2015. Specimens were collected on fruits and leaves of the trees. Insects at all stages of development were collected (nymphs and adult females) and taken to the laboratory for examination. Nymphs were reared until adulthood on persimmon fruits. Labeled specimens were stored in 95% ethyl alcohol.

### Molecular characterization

DNA characterization was performed using the nondestructive method described in Malausa et al. (2011). The DNA region studied was a ~ 760 bp fragment within the mitochondrial region of Cytochrome Oxidase Subunit I previously used in molecular studies on mealybugs (Malausa et al. 2011; Pacheco da Silva et al. 2014). DNA was extracted using the Qiagen DNEasy Tissue kit, following the manufacturer’s recommendations. For amplification were used the primers 5’ CCTTCAACTAATCATAAAAATATYAG 3’ (Forward) and 5’ TAAACTTCTGGATGTCCAAAAAATCA 3’ (Reverse) PCR was performed using the Qiagen Multiplex PCR kit (QIAGEN, Valencia, CA), with a 23 mL reaction mixture and 2 ml of diluted DNA (1–20 ng of DNA matrix). PCR conditions were as follows: initial denaturation at 95 °C for 15 mn; 35 cycles of denaturation at 95 °C for 30 s, hybridization at 48 °C for 90 s, elongation for 60 s; and final extension at 72 °C for 10 mn. PCR-amplified fragments were analyzed with the QIAxcel Advanced System with QIAxcel DNA Fast Analysis cartridges (QIAGEN). PCR products were sent to Beckman Genomics (Takeley, United Kingdom) for bidirectional sequencing on ABI automatic sequencers (Applied Biosystems, Foster City, CA, USA). Consensus sequences and alignments were generated and checked with Bioedit version 7.01. We carried out BLAST searches (MEGABLAST method) on the NCBI GenBank database (http://www.ncbi.nlm.gov/BLAST).

The DNA results are shown for each species after the morphological descriptions. Additionally, all sequence data are available as Suppl. material 1 (FASTA format).

### Morphological identification

The DNA voucher specimens plus other preserved adult females were slide-mounted and identified by light microscopy in the Plant Protection Department of Çukurova University, Adana, Turkey and ANSES, *Laboratoire de la Santé des Végétaux*, Montferrier-sur-Lez, France, according to a slightly modified version of the method of Kosztarab and Kozár (1988). The mealybugs were examined under a LEICA DM 2500 phase-contrast compound microscope and identified with the keys of Williams and Granara de Willink (1992), Gimpel and Miller (1996) and Kaydan and Gullan (2012).

The slides are stored in the Coccoidea Collection of the Museum Ramiro Gomes Costa (MRGC), Porto Alegre, Brazil (Holotype and some paratypes), Çukurova University Coccomorpha collection, Adana, Turkey (KPTC) and Anses, Laboratoire de la Santé des Végétaux, Montferrier-sur-Lez, France (ANSES/LSV).

### Morphometric analysis

Mealybugs were measured and the main taxonomic characters evaluated and quantified under the Leica microscope. Measurements were taken from all the available material. The morphological terms used here are those used by Williams (2004) and Williams and Granara de Willink (1992). All the measurements given are the maximum dimensions (e.g. body width was recorded at the widest part) and are expressed as ranges. Tarsal length excludes the claw. Setal length includes the setal base. Cerarii are numbered as described by Williams and Granara de Willink (1992), with cerarius 1 on the head, anterior to the antenna, and cerarius 17 being on segment VIII.

Illustrations are provided for each species. Each figure represents a generalized individual based on several of the specimens used for description. Each illustration is split longitudinally, with the left half representing the dorsum and the right half the venter. Structural details are shown as enlargements around the central drawing, and are not drawn to the same scale. The translucent pores on the hind legs are mostly found on the dorsal surface, but they are illustrated ventrally on the main figure for convenience. The illustrations and description were prepared by MBK and VCPS.

## Results and discussion

Key to identification of Pseudococcinae genera occurring on fruit trees and in vineyards in Brazil, adapted from Williams (2004) and Williams and Granada de Willink (1992).

**Table:** 

1	Dorsal tubular ducts large, each with an orifice surrounded by a round, sclerotized area containing 1 or more setae within its borders, or with the setae adjacent to the rim	***Ferrisia* Fullaway**
–	Dorsal tubular ducts, if present, without this combination of characters	**2**
2	Dorsal tubular ducts each with a small adventitious pore or cell adjoining the main orifice; anal lobe cerarii each with 7-20 conical setae on a sclerotized area; multilocular disc pores always absent	***Anisococcus* Ferris**
–	Not with this combination of characters; if there are any pores next to tubular ducts, then each anal lobe cerarius usually with only 2 conical setae; multilocular disc pores present or absent	**3**
3	Oral rim tubular ducts present somewhere on the body	**4**
–	Oral rim tubular ducts absent	**6**
4	Dorsal surface with setae on posterior segments at least, each broadly lanceolate or conical in shape, sometimes subequal in size and shape to posterior cerarian setae.	***Nipaecoccus* Sulc**
–	Dorsal surface with all setae flagellate, normally much thinner than cerarian setae	**5**
5	Cerarii anterior to the anal lobe pair mostly with auxiliary setae; with 12–17 distinct pairs of marginal cerarii	***Pseudococcus* Westwood**
–	Cerarii anterior to the anal lobe pair without auxiliary setae; with 1–7 distinct pairs of marginal cerarii	***Maconellicoccus* Ezzat**
6	With 18 distinct pairs of marginal cerarii; anal lobe bars present	***Planococcus* Ferris**
–	With 6 to 17 pairs pairs of marginal cerarii; anal lobe bars present or absent	***Dysmicoccus* Ferris**

### Genus Anisococcus

Taxon classificationAnimaliaHemipteraPseudococcidae

Ferris

Anisococcus Ferris, 1950

#### Type species.

*Dactylopius crawii* Coquillet by original designation.

#### Generic diagnosis

(adapted from Williams and Granara de Willink 1992; McKenzie 1967). Body narrowly to broadly oval, 2.0–3.8 mm long, 1.1–2.8 mm wide. Labium with three segments, about as long as the clypeolabral shield. Antennae, 8-segmented. Circulus present or absent. Legs well-developed, without translucent pores; apparently with a small denticle on the claw. Both ostioles well developed. Anal lobes well developed. Anal ring rounded, usually large and cellular with six long setae, but sometimes reduced, non-cellular, more or less removed from the posterior apex of the abdomen (*Anisococcus ephedrae* (Coquillett)).

*Dorsum*. Dorsal tubular ducts with or without a rim, each orifice associated with one or more minute discoidal pores. Cerarii 13–17 pairs. Anal lobe cerarii, each with 7–20 conical setae on a sclerotized area, often with 3–7 auxiliary setae, remaining cerarii smaller, each with two or more conical setae plus an associated cluster of trilocular pores. Preocular cerarius always absent. Dorsal setae, slender and flagellate. Trilocular pores evenly distributed. Discoidal pores scattered and associated with tubular ducts, each smaller than trilocular pores. Multilocular disc pores absent.

*Venter*. Body setae flagellate. Trilocular pores evenly distributed. Discoidal pores scattered or associated with tubular ducts. Multilocular disc pores absent. Oral collar tubular ducts of one or more sizes, of various lengths and widths, with largest ducts, when present, on body margin, often associated with minute discoidal pores.

#### Key to adult females of *Anisococcus* found in the Neotropical Region

(adapted from Williams and Granada de Willink (1992)).

**Table:** 

1	Dorsal oral collar tubular ducts of one size, all large, each about twice the diameter of a trilocular pore, always with a rim	***Anisococcus milleri* Williams & Granara de Willink**
–	Dorsal oral collar tubular ducts of two sizes, the large ducts with a rim, smaller ducts without a rim	**2**
2	Ventral oral collar tubular ducts present in rows across medial areas of abdominal segments	**3**
–	Ventral oral collar tubular ducts on abdomen represented by only 1 or 2, restricted to medially on abdominal segments	***Anisococcus parasitus* Williams & Granara de Willink**
3	Oral collar tubular ducts on venter of one size; smaller oral collar tubular ducts on dorsum without a sclerotized area next to duct opening	***Anisococcus erbi* Williams & Granara de Willink**
–	Oral collar tubular ducts on venter of two sizes; smaller oral collar tubular ducts on dorsum with a sclerotized area next to duct opening	***Anisococcus granarae* Pacheco da Silva & Kaydan, sp. n.**

### Anisococcus granarae

Taxon classificationAnimaliaHemipteraPseudococcidae

Pacheco da Silva & Kaydan sp. n.

http://zoobank.org/AB5F7C82-5263-4377-97C7-F98E9FC434F6

Figs 1 , 2

#### Type-locality.

**Brazil**, Farroupilha – Rio Grande do Sul, on fruits in persimmon orchards, *Diospyros kaki*, Apr 2015, VC Pacheco da Silva leg.

#### Type-specimen.

*Holotype* female, Brazil, Farroupilha – Rio Grande do Sul, on *Diospyros kaki*, on fruits, Apr 2015, coll: VC Pacheco da Silva, MRGC: 2263. *Paratypes*: Brazil, 3 ♀♀ (85, 84, 89) - Farroupilha – Rio Grande do Sul, on *Diospyros kaki* ‘Fuyu’, Apr 2015, coll: VC Pacheco da Silva and ECW Galzer; 1 ♀ (65) - Bento Gonçalves – Rio Grande do Sul, on *Diospyros kaki*, May 2015, coll: VC Pacheco da Silva; 2 ♀♀ (112, 114) - Farroupilha – Rio Grande do Sul, on *Diospyros kaki* ‘Kioto’, Apr 2015, coll: VC Pacheco da Silva and ECW Galzer; 2 ♀♀ (129, 131) - Caxias do Sul – Rio Grande do Sul, on *Diospyros kaki* ‘Fuyu’, Apr 2015, coll: VC Pacheco da Silva and ECW Galzer; 1 ♀ (142) - Farroupilha – Rio Grande do Sul, on *Diospyros kaki* ‘Kioto’, Apr 2015, coll: VC Pacheco da Silva and ECW Galzer; 1 ♀ (166) - Farroupilha – Rio Grande do Sul, on *Diospyros kaki* ‘Kioto’, Apr 2015, coll: VC Pacheco da Silva and ECW Galzer; 3 ♀♀ (190, 191, 192) - Farroupilha – Rio Grande do Sul, on *Diospyros kaki* ‘Kioto’, Apr 2015, coll: VC Pacheco da Silva and ECW Galzer. ANSES/LSV 3 slides, MBK 2 slide and MRGC 2 slides (2264 and 2265).

#### Diagnosis.

*Anisococcus granarae* Pacheco da Silva & Kaydan, sp. n. is characterized by the following combination of features: (i) dorsal oral collar tubular ducts of 2 sizes, the large type with an indistinct rim, the small type without a rim (but with a sclerotized area next to the ducts opening); (ii) ventral oral collar tubular ducts of two sizes, smaller ducts present in rows across medial areas of abdominal segments, and larger ducts in body margin.

#### Description.

Adult female.

Appearance in life.

**Figure 1.**
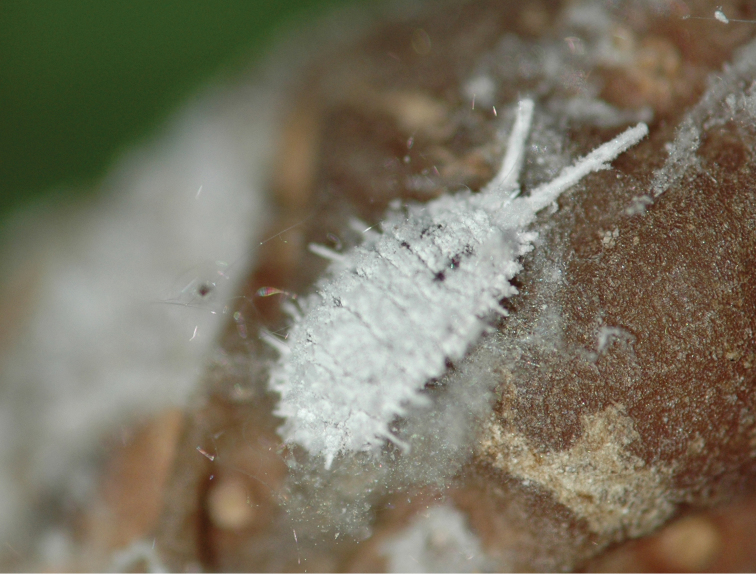
*Anisococcus granarae* Pacheco da Silva & Kaydan, sp. n. Live adult female.

Body oval, up to 4 mm long at maturity, covered in a layer of white wax; with two longitudinal lines of dorsal patches without wax on the intersegmental areas of the abdomen, exposing areas of dark gray-to-black subcutaneous pigment (Fig. 1). The margins have 14 small thin lateral filaments plus a long filament produced by anal lobe cerarii.

**Figure 2.**
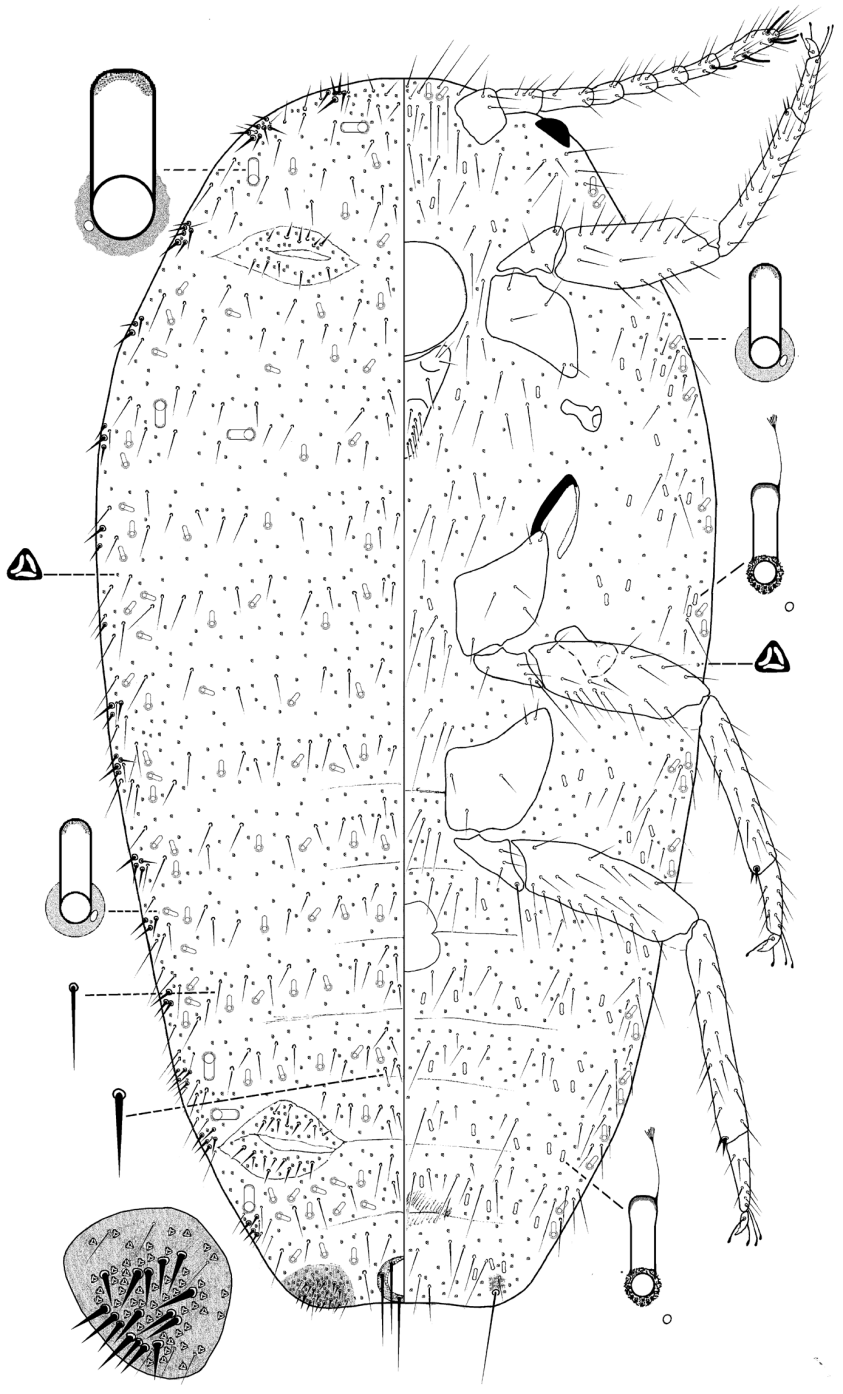
Adult female *Anisococcus granarae* Pacheco da Silva & Kaydan, sp. n. Holotype.

Body oval, 2.08–3.28 mm long, 1.06–1.82 mm wide. Eye marginal, 60–80 μm wide. Antennae, 8-segmented, 630–730 μm long, with 4 fleshy setae, each 35–70 μm long; apical segment 120–125 μm long, 35 μm wide, with apical setae 60 μm long. Tentorium 190–200 μm long, 175–210 μm wide. Labium 3-segmented, 220–260 μm long, 135–145 μm wide. Anterior spiracles 95–105 μm long, 50–65 μm wide across atrium; posterior spiracles 115–130 μm long, 70–90 μm wide across atrium. Circulus 145–200 μm wide. Legs well-developed; lengths for posterior legs: coxa 280–330 μm, trochanter + femur 490–560 μm, tibia + tarsus 550–590 μm, claw 35–45 μm. Ratio of length of tibia + tarsus to trochanter + femur, 1.03–1.15:1; ratio of length of tibia to tarsus, 3.00–3.38:1; ratio of length of hind trochanter + femur to greatest width of femur, 3.25–3.77:1. Tarsal digitules capitate, each 60.0–72.5 μm long. Claw digitules capitate, each 45–50 μm. Both pairs of ostioles present; anterior ostioles each with a total for both lips of 55–69 trilocular pores and 25–30 setae; posterior ostioles each with a total for both lips of 49–69 trilocular pores and 17–22 setae. Anal ring 120–125 μm wide, with 6 setae, each setae 260–305 μm long.

*Dorsum*. Derm membranous, with 16 pairs of cerarii around body margin, each cerarius with 1–6 cerarian setae, each 20.0–22.5 μm long, plus 15–20 trilocular pores between cerarian setae and 3–5 spine-like auxiliary setae. Anal lobe cerarii each with about 12–16 conical setae, each 25.0–32.5 μm long, plus 42–54 trilocular pores and 3–5 spine-like auxiliary setae, all on a sclerotized area about the same size as the anal ring. Dorsal body setae of two kinds, (i) short spine-like slightly flagellate setae, each 20–25 μm long, present in middle of body segments, and (ii) hair-like flagellate setae, each 20–50 μm long, scattered on head and thorax and in single rows on abdominal segments. Trilocular pores each 4–5 μm in diameter, scattered over entire body. Minute discoidal pores, each 2.0–2.5 μm in diameter, also scattered throughout the dorsum and associated with oral collar tubular ducts. Oral collar tubular ducts of two kinds, always with at least 1 minute discoidal pore: (i) larger ducts each 20–25 μm long, 9–10 μm wide at mid-width and with an indistinct rim of duct opening 15 μm wide; totaling 14–21 on the dorsum, with 4 on head, 4 or 5 on thorax and on abdominal segments as follows: II 0–2, III 0–2, IV 0–2, V 2, VI 2, VII 2 and (ii) smaller ducts, each duct 10–15 μm long, 4–5 μm wide at mid-width, with sclerotized area next to duct opening 7.0–7.5 μm wide; scattered throughout on head and thorax, and on abdominal segments as follows: I 12–25, II 12–18, III 14–21, IV 11–21, V 9–13, VI 2–6, VII 25–29, VIII 10–14.

*Venter*. Setae flagellate, each 12.5–225 μm long, longest setae medially on head. Apical setae of anal lobe each 295–360 μm long. Trilocular pores, each 3–4 μm in diameter, frequent throughout the venter. Minute discoidal pores scattered throughout the venter, generally associated with oral collar tubular ducts, each 2–2.5 μm. Oral collar tubular ducts of two sizes: (i) larger ducts concentrated on body margin (same size those on smaller oral collar tubular ducts on dorsum) (2–5 on each side), and (ii) small ducts, each 10.0–12.5 μm long, 2.5–3.0 μm wide, present on head and thorax, and across abdominal segments as follows: I–III 22–31, IV 7–14, V 12–14, VI 6–12, VII 8–10, VIII + IX 0–2.

#### Comments.

*Anisococcus granarae* Pacheco da Silva & Kaydan sp. n. is most similar to *Anisococcus erbi* Williams & Granara de Willink and *Anisococcus parasitus* Williams & Granara de Willink in having oral collar tubular ducts of two sizes on the dorsum. However, *Anisococcus granarae* can be readily distinguished from *Anisococcus erbi* in having: (i) oral collar tubular ducts of two sizes on the venter, and (ii) 16 cerarii on body margins (13 –15), and from *Anisococcus parasitus* in having: (i) oral collar tubular ducts of two sizes on the venter (*Anisococcus parasitus* has oral collar tubular ducts of only one size), and (ii) ventral oral collar tubular ducts present in rows across medial areas of the abdominal segments (not in rows on *Anisococcus parasitius*).

#### Etymology.

This species is named after Dr. Maria Cristina Granara de Willink who carried out the most valuable studies on the systematics and taxonomy of mealybugs in Central and South America.

#### Host plant.

*Diospyros kaki*.

#### Distribution.

Brazil (Bento Gonçalves, Caxias do Sul and Farroupilha, Rio Grande do Sul).

#### Molecular characterization.

No intraspecific variation was observed at COI (35 replicates). No BLAST hit with high similarity (> 95%) was obtained with GenBank.

### Genus Ferrisia

Taxon classificationAnimaliaHemipteraPseudococcidae

Fullaway

Ferrisia Fullaway, 1923
Ferrisiana Takahashi, 1929

#### Type species.

*Dactylopius virgatus* Cockerell, by monotypy and original designation.

#### Generic diagnosis

(adapted from Kaydan and Gullan 2012). Adult female. Body elongate to oval, 1.3–5.5 mm long, 0.5–3.0 mm wide. Antennae almost always 8-segmented (sometimes 7-segmented in *Ferrisia milleri* Kaydan & Gullan and *Ferrisia pitcairnia* Kaydan & Gullan). Labium 3-segmented, always longer than wide. Posterior pair of spiracles always larger than anterior spiracles. Circulus quadrate, divided by an intersegmental line. Legs well-developed, with or without translucent pores on hind coxa, femur and tibia; claw without a denticle; tarsal and claw digitules both capitate, claw digitules thicker than tarsal digitules. Posterior ostioles well-developed; anterior ostioles usually more weakly developed than posterior pair, or absent. Anal lobes well developed. Anal ring typically with 6 anal ring setae.

#### Description.

*Dorsum*. With long enlarged ducts, each with the orifice surrounded by a circular sclerotized rim, either containing short setae or with setae just outside border. In living insects, these ducts secrete long glassy filaments typical of the genus. Cerarii confined to anal lobes; each anal lobe usually with 2 enlarged conical setae (more on some specimens of *Ferrisia dasylirii* Cockerell and *Ferrisia virgata* (Cockerell)) plus an associated cluster of trilocular pores and a few auxiliary setae. Body setae slender and flagellate, bluntly tipped to slightly capitate, and of various sizes. Trilocular pores each 3–5 μm in diameter, often slightly larger (4–5 μm diameter) than ventral trilocular pores (typically 3–5 μm), scattered over the dorsum. Minute discoidal pores on the dorsal submargin of the head at base of antennal segment I, usually in a small tight cluster of 3–8 pores (often difficult to see), and also associated with enlarged tubular ducts (generally present within sclerotized area surrounding duct rim). Enlarged tubular ducts present mostly on body margin and submargin in segmental clusters, but often also present medially and submedially; duct opening of each tubular duct with a sclerotized rim surrounded by a circular sclerotized area bearing 0–3 (generally 1 or 2) minute discoidal pores (appearing as clear areas in the cuticle) and with 1–7 (generally 3–5) blunt-tipped to slightly capitated setae. Oral-collar tubular ducts and multilocular pores absent.

*Venter*. Body setae slender, blunt-tipped to slightly capitate, and of various sizes. Trilocular pores each 2.5–5.0 μm in diameter, scattered over surface. Minute discoidal pores scattered throughout the venter, almost always associated with ventral oral-collar tubular ducts. Enlarged tubular ducts absent. Oral-collar tubular ducts of one or more sizes, of various lengths and widths, shortest ducts often present in marginal clusters, at least on posterior abdominal segments; ducts on anterior abdomen and margins or submargins of posterior abdomen often associated with a minute discoidal pore (rarely 2 pores), usually appearing as a clear circular to oval area in cuticle. Multilocular disc pores generally present (absent in *Ferrisia meridionalis* Williams) on posterior abdominal segments, especially around the vulva.

#### Key to adult females of *Ferrisia* from the Neotropical Region

(adapted from Kaydan and Gullan (2012)). The key includes only species displaying the following combination of features: (i) ventral oral-collar tubular ducts of at least 2 sizes, smaller ducts present singly or in segmental clusters on the body margin, at least on the last 2 or 3 abdominal segments, and (ii) minute discoidal pores in sclerotized area of enlarged tubular ducts, touching the sclerotized rim of the duct opening.

**Table:** 

1	Translucent pores absent from hind coxae; each anal lobe with ≥60 trilocular pores; small oral-collar tubular ducts usually in tight segmental clusters on ventral margins of posterior 2 or 3 abdominal segments, distributed 0–7 on each side of segment VI, 6–25 on each side of VII, and 8–21 on each side of VIII	***Ferrisia kondoi* Kaydan & Gullan**
–	Translucent pores present on each hind coxa, >20 in number; each anal lobe with ≤50 trilocular pores; small oral-collar tubular ducts on ventral margins of posterior 2 or 3 abdominal segments either not forming tight clusters or, if perhaps in clusters, these are small, each segment usually with ≤6 ducts on each side	**2**
2	Ventral oral-collar tubular ducts on abdominal submargin (not those in posterior marginal clusters) sometimes with 2 contiguous elliptical to elongate triangular discoidal pores in sclerotized rim of duct (check with 100× objective)	***Ferrisia williamsi* Kaydan & Gullan**
–	Ventral oral-collar tubular ducts on abdominal submargin (not those in posterior marginal clusters) with a circular discoidal pore in sclerotized rim of duct or on nearby derm in at least some ducts	**3**
3	Multilocular disc pores only on abdominal segments VII and VII+IX; 87-99 enlarged tubular ducts present on dorsum; translucent pores on hind legs totaling 16–31 on all segments combined; with 11–15 on each hind coxa; small oral collar tubular ducts on last ventral abdominal segments numbering 1–3 on each side of VII; 0–1 on each side of VIII+IX	***Ferrisia kaki* Kaydan & Pacheco da Silva, sp. n.**
–	Multilocular disc pores only on abdominal segments VI and VII+IX; 95-113 enlarged tubular ducts on dorsum; translucent pores on hind legs totaling 80–93 on all segments combined; with 22–55 on each hind coxa; small oral collar tubular ducts on last ventral abdominal segments numbering 3–6 on each side of VII; 3–6 on each side of VIII+IX	***Ferrisia cristinae* Kaydan & Gullan**

### Ferrisia kaki

Taxon classificationAnimaliaHemipteraPseudococcidae

Kaydan & Pacheco da Silva sp. n.

http://zoobank.org/47CFCF98-E37E-40BB-B7DD-634F75242FA1

Fig. 3

#### Type-locality.

Brazil, Caxias do Sul – Rio Grande do Sul, on fruits in persimmon orchards, *Diospyros kaki*, Apr 2015, VC Pacheco da Silva leg.

#### Type-specimen.

*Holotype* female, Brazil, Caxias do Sul – Rio Grande do Sul, on *Diospyros kaki*, on fruits, Apr 2015, coll: VC Pacheco da Silva, MRGC: 2266. *Paratypes*: Brazil, 4 ♀♀ Caxias do Sul – Rio Grande do Sul, on *Diospyros kaki* ‘Fuyu’, iv.2015, coll: VC Pacheco da Silva and ECW Galzer; 1 ♀ Farroupilha – Rio Grande do Sul, on *Diospyros kaki* ‘Kioto’, iv.2015, coll: VC Pacheco da Silva and ECW Galzer. ANSES/LSV 1 slide, MBK 3 slides and MRGC 1 slide (2267).

**Figure 3.**
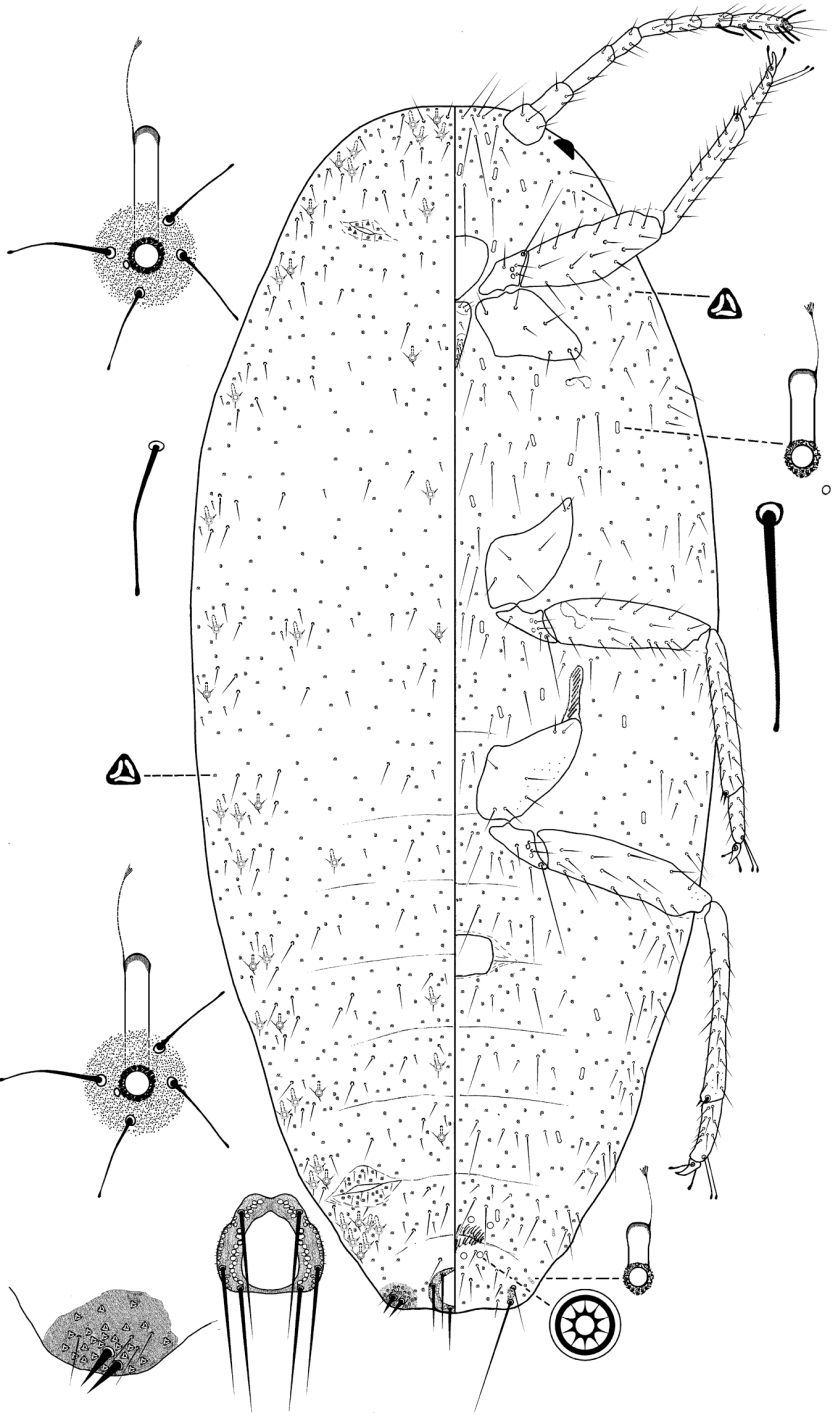
Adult female *Ferrisia kaki* Kaydan & Pacheco da Silva, sp. n. Holotype.

#### Diagnosis.

*Ferrisia kaki* Kaydan & Pacheco da Silva, sp. n. is characterized by the following combination of features: (i) ventral oral-collar tubular ducts of two sizes, smaller ducts present singly or in segmental clusters on the body margin, on the last two or three abdominal segments; (ii) minute discoidal pores on the sclerotized area of enlarged tubular ducts, almost always touching the sclerotized duct rim, and (iii) both anterior and posterior pairs of ostioles present and well-developed.

#### Description.

Adult female.

Appearance in life is unrecorded.

Body oval, 2.76–3.74 mm long, 1.26–1.78 mm wide. Eye marginal, 60–70 μm wide. Antennae 8-segmented, 650–700 μm long, with 4 fleshy setae, each 30–55 μm long; apical segment 125–130 μm long, 35.0–37.5 μm wide, apical setae 35–45 μm long. Clypeo-labral shield 160–195 μm long, 135–195 μm wide. Labium 3-segmented, 205–215 μm long, 115–130 μm wide. Anterior spiracles 70–75 μm long, 35–45 μm wide across atrium; posterior spiracles 75–85 μm long, 50–60 μm wide across atrium. Circulus 125–130 μm wide. Legs well-developed; length of posterior legs: coxa 260–300 μm, trochanter + femur 470–500 μm, tibia + tarsus 520–570 μm, claw 37–43 μm. Ratio of length of tibia + tarsus to trochanter + femur, 1.06–1.19:1; ratio of length of tibia to tarsus, 2.82–3.14:1; ratio of length of hind trochanter + femur to greatest width of femur, 3.91–4.70:1. Translucent pores present on the coxa (11–15), femur (3–7) and tibia (2–8). Tarsal digitules capitate, each 55–60 μm long. Claw digitules capitate, each 32–45 μm. Both pairs of ostioles present; anterior ostioles each with a total for both lips of 29–32 trilocular pores and 10–12 setae; posterior ostioles each with a total for both lips of 12–16 trilocular pores and 3–5 setae. Anal ring 100–110 μm wide, with 6 setae, each setae 170–193 μm long.

*Dorsum*. Derm membranous, with only anal lobe pairs of cerarii, each cerarius with 2 cerarian setae, each 30–35 μm long, plus 28–41 trilocular pores between cerarian setae and 3–5 hair-like auxiliary setae. Dorsal body setae hair-like, flagellate, blunt, each 12.5–60.0 μm long, scattered on head and thorax, and in single rows on abdominal segments. Trilocular pores, each 3–4 μm in diameter, scattered over entire body. Minute discoidal pores each 2.0–2.5 μm in diameter, scattered all over body and also associated with enlarged tubular ducts, almost always touching the sclerotized duct rim. Enlarged tubular ducts, each 35.0–42.5 μm long, 6.5–7.5 μm wide at mid-width; rim of duct opening 8–10 μm wide, sclerotized area 20–30 μm wide, bearing 2–7 hair-like setae, each 15–35 μm long; with 87–99 in total, present on head and thorax, and each side of the abdominal segments and also medially on segments IV-VI, numbering as follows: I 1 or 2, II 1 or 2, III 2, IV 1 or 2, V 2 or 3, VI 2 or 3, VII 6–8.

*Venter*. Setae flagellate, blunt, each 12.5–210 μm long, longest setae medially on head. Apical setae of anal lobe each 280–300 μm long. Multilocular disc pores each 7–9 μm in diameter, in rows on abdominal segments, as follows: VII 2–4, VIII + IX 4. Trilocular pores, each 3–4 μm in diameter, scattered throughout on the venter. Minute discoidal pores, each 2–2.5 μm wide, scattered throughout and associated with oral collar tubular ducts. Oral collar tubular ducts of two sizes, (i) smaller ducts, each 6.5–7.5 μm long, 3 μm width, present on each side of body margin of abdominal segments, as follows: VI 1, VII 1–3, VIII+IX 0–1, and (ii) larger ducts, each14–16 μm long, 3 μm wide, sparse on head and thorax and across abdominal segments, as follows: III 1 or 2, IV 2 or 3, V 2 or 3, VI 2 or 3, VII 2–4, VIII + IX 0.

#### Comments.

*Ferrisia kaki* Kaydan & Pacheco da Silva, sp. n. most closely resembles *Ferrisia cristinae* Kaydan & Gullan, in having few ventral oral-collar tubular ducts on the abdominal submargin (not those in posterior marginal clusters), and often with a circular discoidal pore in the sclerotized rim of the duct or on nearby derm. However, *Ferrisia kaki* differs from *Ferrisia cristinae* in having: (i) multilocular disc pores only on abdominal segments VII and VIII+IX (VI–VIII+IX in *Ferrisia cristinae*) and (ii) 87–99 enlarged tubular ducts on the dorsum (95–113 in *Ferrisia cristinae*). *Ferrisia kaki* is also similar to *Ferrisia terani* Williams in having a small number of multilocular disc pores and a slender body shape, but *Ferrisia kaki* can be readily distinguished from *Ferrisia terani* in having: (i) two sizes of oral collar tubular ducts on the venter (only one size in *Ferrisia terani*); (ii) enlarged tubular ducts with a minute discoidal pore touching the sclerotized rim of duct opening.

#### Etymology.

This species was named after its host plant, to reflect the high levels of infestation in persimmon orchards.

#### Host plant.

*Diospyros kaki*.

#### Distribution.

Brazil (Caxias do Sul, Farroupilha, Rio Grande do Sul).

#### Molecular characterization.

**Figure 4.**
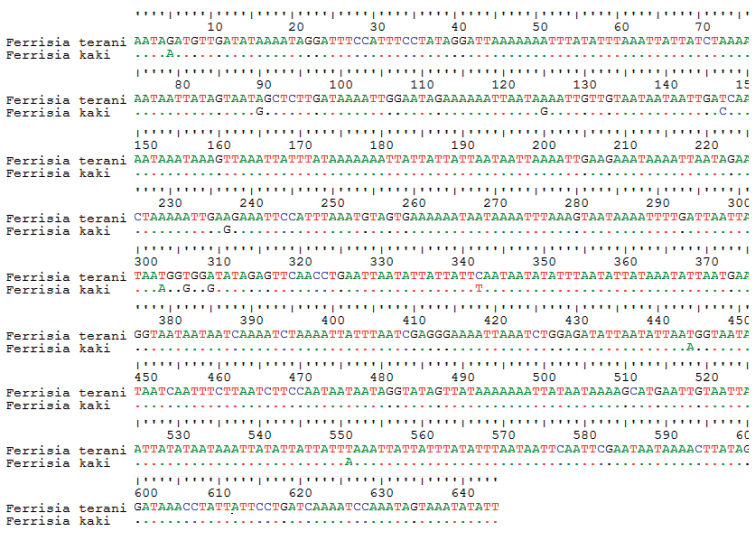
Alignment of the COI DNA sequences obtained for *Ferrisia kaki* Kaydan & Pacheco da Silva, sp. n. and *Ferrisia terani* (from Pacheco da Silva et al. 2014). *Ferrisia terani* is used as reference sequence in the alignment and only the differences to this reference are displayed in the sequence of *Ferrisia kaki*.

No intraspecific variation was observed at COI (7 replicates). A BLAST hit with sequence similarity of 98% was obtained with a sequence assigned to *Ferrisia terani* Williams & Granara de Willink from Pacheco da Silva et al. (2014). The alignment between *Ferrisia kaki* and *Ferrisia terani* is shown in Figure 4.

### Genus Pseudococcus

Taxon classificationAnimaliaHemipteraPseudococcidae

(Westwood)

Pseudococcus (Westwood), 1840
Trechocorys Curtis, 1843
Boisduvalia Signoret, 1875
Oudablis Signoret, 1882

#### Type species.

*Dactylopius longispinus* Targioni Tozzetti.

#### Generic diagnosis

(adapted from Gimpel and Miller 1996; Williams and Granara de Willink 1992). Adult female. Body normally broadly oval, 1.2–4.3 mm long, 0.6–2.6 mm wide. Antennae each normally 8-segmented, occasionally with 7 segments. Labium 3-segmented, always longer than wide. Legs well-developed, claw without a denticle; translucent pores generally present on hind legs, on coxae, and/or femora and/or tibiae, rarely on trochanter; tarsal and claw digitules both capitate, claw digitules thicker than tarsal digitules. Circulus usually present, well-developed and divided by an intersegmental line; rarely small and not divided, usually wider than long. Quinquelocular pores always absent.

#### Description.

*Dorsum*. Dorsal setae flagellate. Anterior and posterior ostioles present, well-developed. Cerarii present, 12–17 pairs, preocular pair always absent, each cerarius normally with two conical setae, except for 1 or 2 on head and thorax, each often with 3 or 4 conical setae plus an associated cluster of trilocular pores; anal lobe cerarii well-developed, each often sclerotized, usually with two enlarged conical setae; usually all cerarii with auxiliary setae, but occasionally auxiliary setae absent anterior to the penultimate pair. Anal ring typically with six setae. Trilocular pores scattered over dorsum. Minute discoidal pores usually present, sometimes situated adjacent to rim of oral rim tubular ducts. Oral rim tubular ducts present on body margins and medially and submedially, or in rows across abdominal segments, sometimes associated with minute discoidal pores and setae. Oral-collar tubular ducts often present. Multilocular pores rarely present on dorsum.

*Venter*. Body setae flagellate. Trilocular pores scattered over entire surface. Minute discoidal pores scattered throughout the venter, often of two sizes, larger pores frequently present next to eyes and on venter of anal lobes, sometimes also situated adjacent to rim of oral rim tubular ducts. Oral rim tubular ducts occasionally on venter only. Oral-collar tubular ducts of one or more sizes, of various lengths and widths, shortest ducts often present medially on abdominal segments, and larger ducts often present on margins of abdomen. Multilocular disc pores present on posterior abdominal segments, especially around vulva.

#### Key to adult females of the *Pseudococcus maritimus* complex with multilocular disc pores present on dorsum (adapted from Gimpel and Miller (1996)).

**Table:** 

1	Oral collar tubular ducts scattered over dorsum	***Pseudococcus rosangelae* Pacheco da Silva & Kaydan, sp. n.**
–	Oral collar tubular ducts, if present, only on margins or on abdominal segments	**2**
2	Dorsal multilocular disc pores scarce, restricted to segments V-VII; fewer than 10 ventral multilocular disc pores on head and thorax (in total)	**3**
–	Dorsal multilocular disc pores numerous on segments III-VIII; with more than 20 ventral multilocular disc pores on head and thorax (in total)	***Pseudococcus peregrinabundus* Borchsenius**
3	More than 50 translucent pores present on hind tibia; 1–6 large discoidal pores associated with each eye; 137–258 trilocular pores present on segment VI of venter	***Pseudococcus nakaharai* Gimpel & Miller**
–	Fewer than 20 translucent pores present on hind tibia; 0–2 small discoidal pore associated with each eye; 42–54 trilocular pores present on segment VI of venter	***Pseudococcus dasyliriae* Gimpel & Miller**

### Pseudococcus rosangelae

Taxon classificationAnimaliaHemipteraPseudococcidae

Pacheco da Silva & Kaydan sp. n.

http://zoobank.org/D4E09C38-771A-47A2-BACF-8622154C4726

Fig. 5

#### Type-locality.

Brazil, Farroupilha, Rio Grande do Sul, on fruits in persimmon orchards, *Diospyros kaki*, 15 Apr 2015, VC Pacheco da Silva leg.

#### Type-specimen.

*Holotype* female, Brazil, Farroupilha, Rio Grande do Sul, on *Diospyros kaki*, on fruits, Apr 2015, coll: VC Pacheco da Silva, MRGC: 2262.

**Figure 5.**
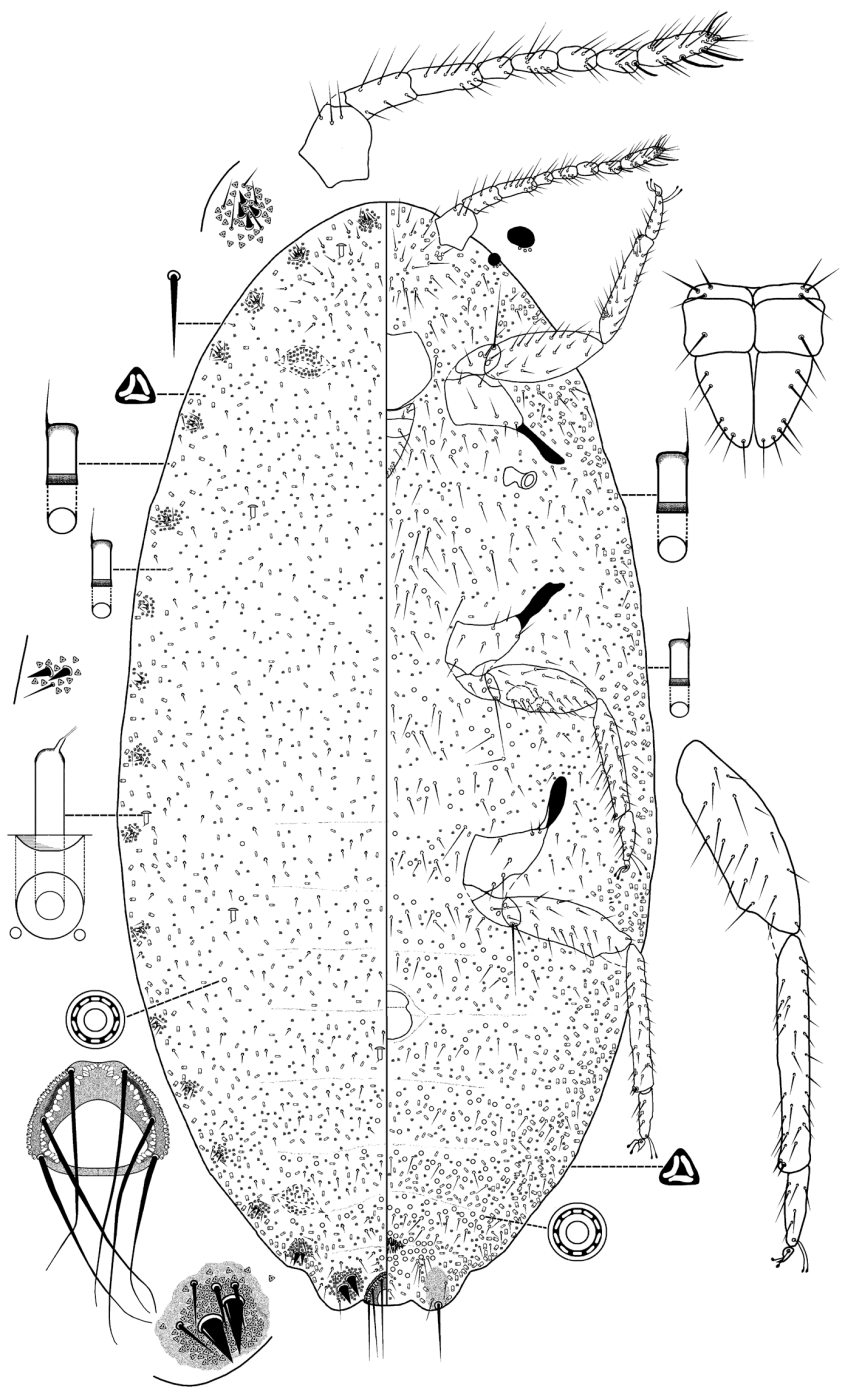
Adult female *Pseudococcus rosangelae* Pacheco da Silva & Kaydan, sp. n. Holotype.

#### Diagnosis.

*Pseudococcus rosangelae* Pacheco da Silva & Kaydan, sp. n. is characterized by the following combination of features: (i) multilocular disc pores present on the dorsum, and (ii) dorsal oral collar tubular ducts scattered throughout.

#### Description.

Adult female. Appearance if life is unrecorded.

Body oval, elongate, 2.72 mm long, 1.32 mm wide. Eye marginal, 40 μm wide, each with 3 discoidal pores. Antennae 8-segmented, 560–565 μm long, with 4 fleshy setae, each 25.0–42.5 μm long; apical segment 102.5 μm long, 32.5–35.0 μm wide, with apical setae 45.0–47.5 μm long. Clypeolabral shield 175 μm long, 202.5 μm wide. Labium 3-segmented, 175 μm long, 122.5 μm wide. Anterior spiracles 75–80 μm long, 45 μm wide across atrium; posterior spiracles 82.5–90 μm long, 55–60 μm wide across atrium. Circulus 125 μm long, 135 μm wide. Legs well-developed; lengths for posterior legs: coxa 245.0–252.5 μm, trochanter + femur 405–410 μm, tibia + tarsus 460–475 μm, claw 37.5–40.0 μm. Ratio of length of tibia + tarsus to trochanter + femur, 1.13–1.16:1; ratio of length of tibia to tarsus, 2.80–2.84:1; ratio of length of hind trochanter + femur to greatest width of femur, 3.72–3.85:1; translucent pores absent on legs. Tarsal digitules capitate, each 50–57.5 μm long. Claw digitules capitate, 35 μm long. Both pairs of ostioles present; anterior ostioles each with a total for both lips of 29–32 trilocular pores and 6 setae; posterior ostioles each with a total for both lips of 35–39 trilocular pores and 3–6 setae. Anal ring 72.5 μm wide, with 6 setae, each seta 140.0–167.5 μm long.

*Dorsum*. Derm membranous, with 17 pairs of cerarii around body margin, each cerarius with 2–4 cerarian setae. Setae on each anal lobe cerarius 25.0–27.5 μm long, 10 μm wide, plus 67–76 trilocular pores and 3–4 spine-like auxiliary setae. Dorsal setae short and flagellate, each 5–20 μm long, scattered throughout the dorsum. Trilocular pores, each 3.7–5.0 μm in diameter, scattered over entire body. A few minute discoidal pores, each 2.5–3.0 μm in diameter, scattered over dorsum. Oral rim tubular ducts, each 12.5 μm long, 3.7 μm wide at mid-width, rim of duct opening 3.7–5.0 μm wide and outer width 7.5–10 μm, seven in total on dorsum, with two ducts on head, two on thorax, and on abdominal segments, as follows: I 2, III 1, IV 1. Oral collar tubular ducts of two sizes: (i) larger ducts, each 5.0 μm long, 3.7–5.0 μm wide, along entire margin of the body; and (ii) smaller ducts, each 5.0–6.2 μm long, 2.5–3.75 μm wide, present throughout the dorsum but in bands on abdominal segments, as follows: I–III 141, IV 88, V 41, VI 32, VII 25, VIII + IX 26. Multilocular disc pores, each 5.0–7.5 μm in diameter, present on abdominal segments, as follows: I–III 6, IV 2, V 6, VI 10, VII 10, VIII + IX 2.

*Venter*. Setae short and flagellate, each 7.5–145 μm long, longest setae located medially on head. Apical setae of anal lobe each132 μm long. Trilocular pores and minute discoidal pores scattered all over body. Trilocular pores, each 3.7–5.0 μm scattered throughout the venter. Oral collar tubular ducts of 2 sizes: (i) larger ducts, each 5.0–6.3 μm long, 3.7–5.0 μm wide in the margin of the body and throughout, and (ii) smaller oral ducts, each 6.2–7.5 μm long, 2.0–2.5 μm wide, present throughout, and also as bands across abdominal segments, as follows: I–III 110, IV 69, V 81, VI 60, VII 47, VIII + IX 23. Multilocular disc pores, each 5.0–7.5 μm in diameter, present throughout on the venter and on the abdominal segments, as follows: I–III 46, IV 15, V 30, VI 41, VII 29, VIII + IX 21.

#### Comments.

*Pseudococcus rosangelae* Pacheco da Silva & Kaydan most closely resembles *Pseudococcus peregrinabundus*, *Pseudococcus nakaharai* and *Pseudococcus dasyliriae* in having dorsal multilocular disc pores, but *Pseudococcus rosangelae* can be distinguished from other species in having: (i) oral collar tubular ducts present over the entire dorsum (on other species not scattered all over the dorsum) and (ii) no translucent pores on the hind legs (present in the other species).

#### Etymology.

This species is named after Rosangela Leme do Prado, mother of the author VCPS.

#### Host plant.

*Diospyros kaki*.

#### Distribution.

Brazil (Farroupilha, Rio Grande do Sul).

#### Molecular characterization.

No DNA sequence was obtained for *Pseudococcus rosangelae*.

## Supplementary Material

XML Treatment for Anisococcus

XML Treatment for Anisococcus granarae

XML Treatment for Ferrisia

XML Treatment for Ferrisia kaki

XML Treatment for Pseudococcus

XML Treatment for Pseudococcus rosangelae

## Supplementary materials

Supplementary material 1
COI DNA sequences obtained for *Anisococcus granarae* Pacheco da Silva & Kaydan, sp. n. and *Ferrisia kaki* Kaydan & Pacheco da Silva, sp. n. Data type: FASTA file Explanation note: This supplementary file contais the senquences of a fragment from the mitochondrial region of Cytochrome Oxidase Subunit I of two new species of mealybugs found on persimmon trees in Southern Brazil, *Anisococcus granarae* Pacheco da Silva & Kaydan, sp. n. and *Ferrisia kaki* Kaydan & Pacheco da Silva, sp. n. This dataset is made available under the Open Database License (http://opendatacommons.org/licenses/odbl/1.0/). The Open Database License (ODbL) is a license agreement intended to allow users to freely share, modify, and use this Dataset while maintaining this same freedom for others, provided that the original source and author(s) are credited.Vitor C. Pacheco da Silva, Mehmet Bora Kaydan, Jean-François Germain, Thibaut Malausa, Marcos Botton
